# Foetal death due to extensive extra-abdominal umbilical vein Varix with umbilical vein thrombosis: a case report

**DOI:** 10.1186/s12884-023-05485-w

**Published:** 2023-03-08

**Authors:** Qing-Yun Song, Ying Tang

**Affiliations:** 1 Department of Diagnostic Ultrasound, West China Second University Hospital, Sichuan University, 610041 Chengdu, Sichuan Province China; 2 Key Laboratory of Obstetric & Gynecologic and Pediatric Diseases and Birth Defects of Ministry of Education, Chengdu, China

**Keywords:** Extra-Abdominal, Umbilical vein Varix, Umbilical, Thrombosis, Foetal death, Ultrasound, MRI, Prenatal diagnosis, Case Report

## Abstract

**Background:**

Foetal anaemia and umbilical vein thrombosis are rare pregnancy complications that can increase the risk of perinatal adverse events, which, in severe cases, can lead to foetal death. During pregnancy, umbilical vein varix (UVV) commonly occurs in the intra-abdominal part of the umbilical vein and is associated with an increased risk of foetal anaemia and umbilical vein thrombosis. However, UVV occurring in the extra-abdominal part of the umbilical vein is rare, especially when accompanied by thrombosis. In this case report, we describe a rare case of an extensive extra-abdominal umbilical vein varix (EAUVV), which ultimately resulted in foetal death due to umbilical vein thrombosis.

**Case presentation:**

In this report, we describe a rare case of an extensive EAUVV that was discovered at 25 weeks and 3 days of gestation. During the examination, there were no abnormalities in foetal haemodynamics. The estimated weight of the foetus was only 709 g. In addition to refusing to be hospitalized, the patient refused close monitoring of the foetus. As a result, we were limited to choosing an expectant therapy. The foetus died 2 weeks after diagnosis and was confirmed to have EAUVV with thrombosis after the induction of labour.

**Conclusion:**

In the case of EAUVV, lesions are extremely rare, and it is very easy for thrombosis to form, which may result in the death of the child. When determining the next step in the treatment of the condition, the degree of UVV, possible complications, gestational age, foetal haemodynamics, and other relevant factors are strongly connected to the clinical therapy decision, and these factors should be considered comprehensively when making a clinical decision. We recommend close monitoring with hospital admission (to facilities capable of handling extremely preterm foetuses) after variability in delivery for worsening haemodynamic status.

**Supplementary Information:**

The online version contains supplementary material available at 10.1186/s12884-023-05485-w.

## Background

Foetal anaemia and umbilical vein thrombosis are rare pregnancy complications that can increase the risk of perinatal adverse events, which, in severe cases, can lead to foetal death [1–5]. Umbilical vein varix (UVV) occurs when abnormal dilatation occurs in the foetal umbilical veins. In most cases, UVVs occur in the intra-abdominal part of the umbilical vein. Approximately 4% of umbilical cord anomalies are associated with this condition, which occurs in 0.4–1.1 out of 1000 pregnancies [3, 6]. However, UVVs occurring in the extra-abdominal part of the umbilical vein are rare, especially when accompanied by thrombosis. In this case report, we describe a rare case of an extensive extra-abdominal umbilical vein varix (EAUVV), which ultimately resulted in foetal death due to umbilical vein thrombosis.

## Case presentation

A 29-year-old woman, G3P1 (gravida 3, para 1), without any relevant medical history, was referred to our hospital for intrauterine growth retardation and umbilical cord oedema at 25 weeks and 3 days of gestation. There were no pregnancy-related complications observed. Ultrasonography using the Hadlock 4 measurements formula revealed that the estimated foetal weight was 709 g (at approximately 24 weeks and 2 days of gestiation, 12.0%). Doppler studies of the umbilical artery and ductus venosus were normal. The peak systolic velocity (PSV) of the foetal middle cerebral artery was 0.91 Mom. The deepest vertical pocket of amniotic fluid was 6.8 cm.

**Fig. 1:**
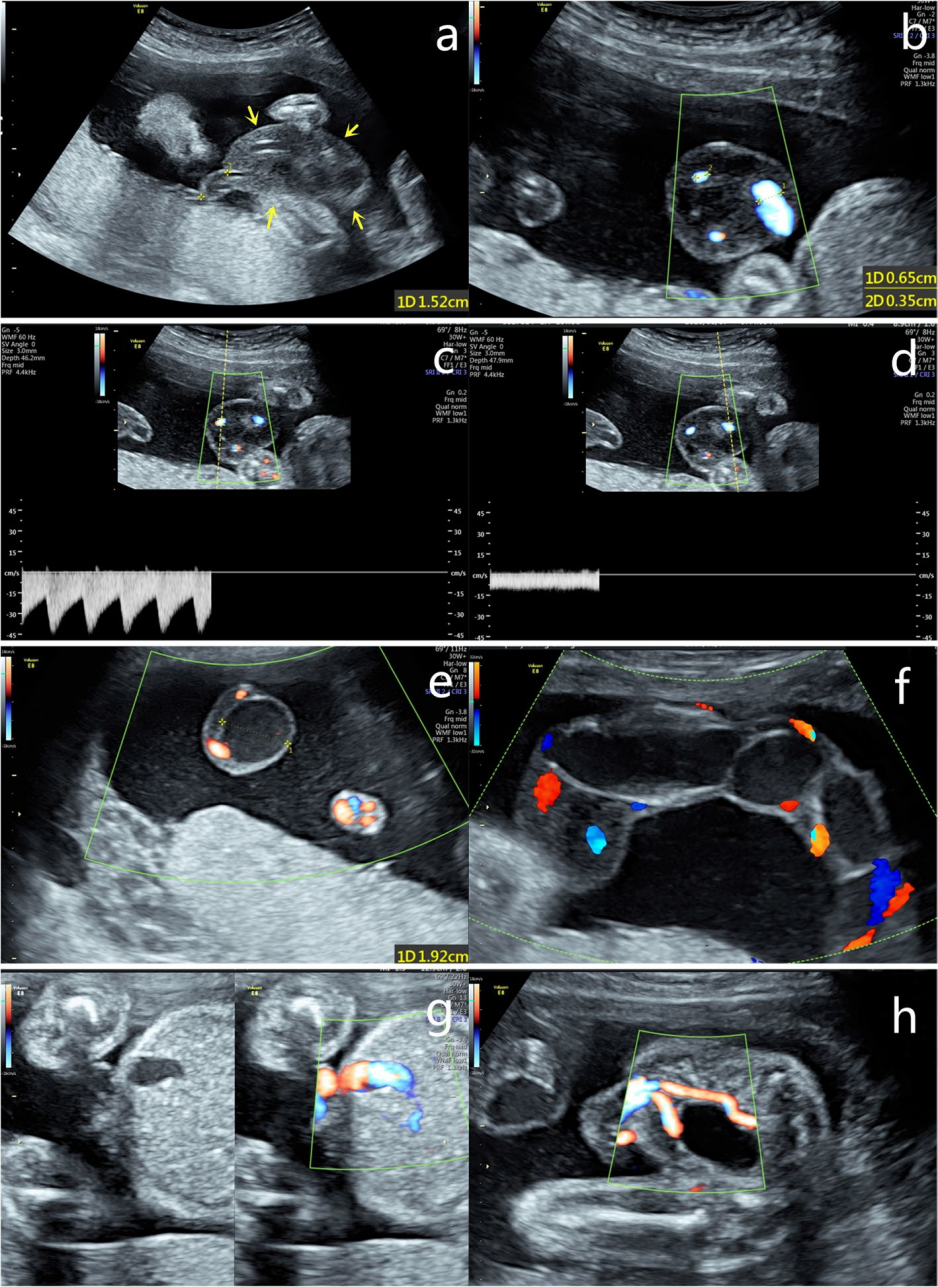
Ultrasonography at 25 weeks of gestation. **a** An approximately 4.2-cm-long thickening of Wharton jelly was noted about 1.5 cm away from the placental cord insertion(arrows), **b** and the cross-sectional view revealed that the thickened Wharton jelly wrapped around three blood vessels: the inner diameter of the umbilical vein was approximately 0.65 cm; the inner diameter of the umbilical artery was approximately 0.35 cm; and pulse Doppler had probed **c** the arterial and **d** venous blood flow spectrums, respectively. **e** The cross-sectional view of the middle part of the umbilical cord revealed only two umbilical arteries with blood flow signals. The umbilical vein was broadened, with an inner diameter of approximately 1.9 cm. **f** The vertical section showed that the broadened umbilical vein was approximately 20 cm long, and the blood flow in it was extremely slow. **g** The inner diameter of the widest part of the intra-abdominal segment of the umbilical vein was approximately 0.74 cm. **h** No abnormality of the umbilical artery was found in the intra-abdominal segment

**Fig. 2:**
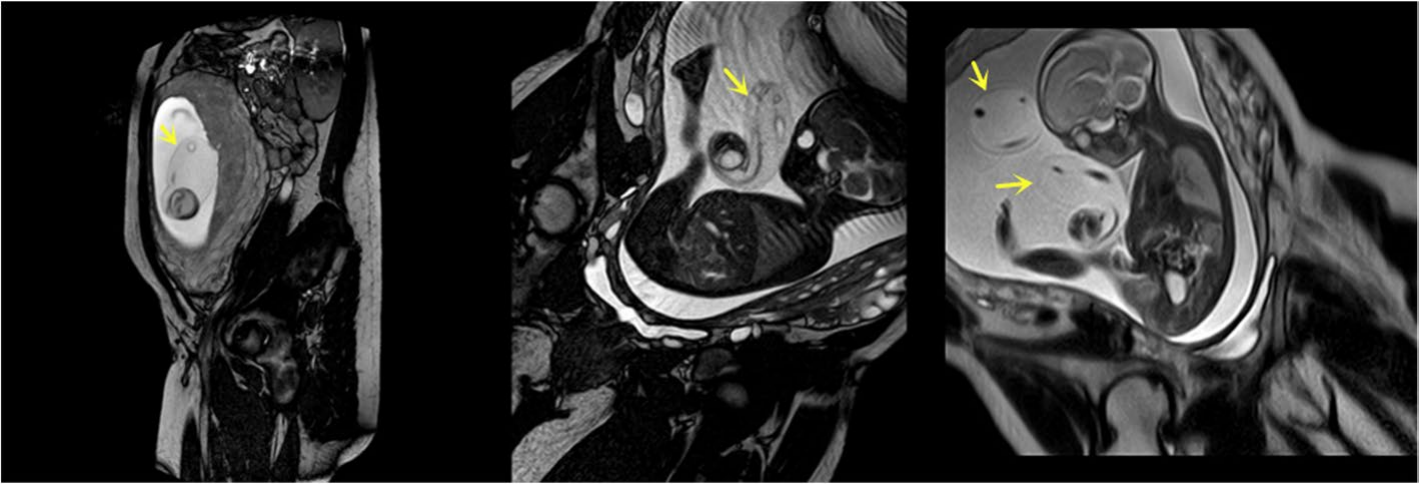
Magnetic resonance imaging (MRI) revealed that the local umbilical vein in the umbilical cord had lost its normal contours and become thick and cystic(arrows)

**Fig. 3:**
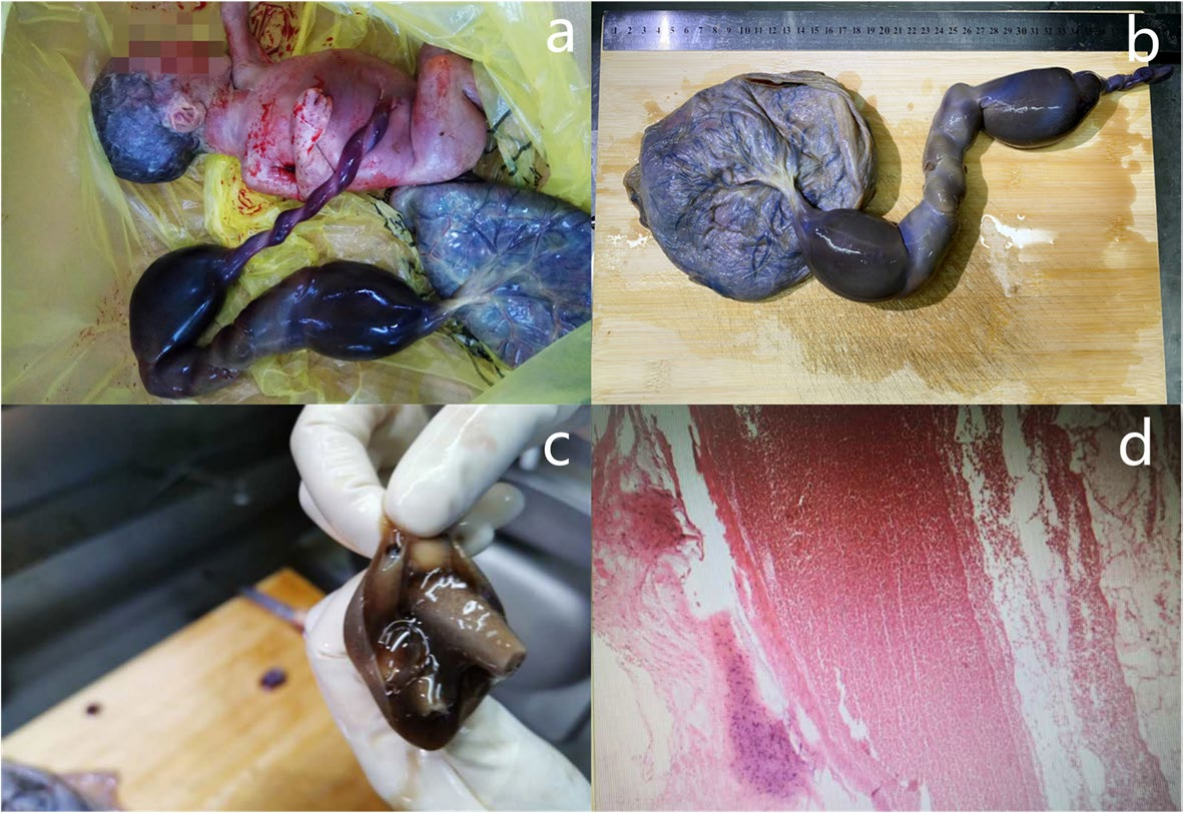
The gross specimen after induction of labor suggested **a** obvious umbilical vein varices, which were mainly near the placenta, **b,c** with a wide range of more than 20 cm long and a widest inner diameter of approximately 6 cm. Histopathological investigations revealed **d** umbilical vein thrombosis

Ultrasonography showed extensive EAUVV (Fig. 1). The enlarged umbilical vein was approximately 20 cm long. Two-dimensional ultrasound suggested blood stasis within the varix (Additional File 1). Magnetic resonance imaging (MRI) revealed that the local umbilical vein in the umbilical cord had lost its normal contours and become thick and cystic (Fig. 2). The patient was informed that there was an EAUVV, blood flow stasis, a very high risk of umbilical vein thrombosis and a very high chance of intrauterine death. Due to the early gestational age of the foetus, the estimated foetal weight was only 709 g. In addition to refusing to be hospitalized, the patient refused close monitoring of the foetus. Therefore, we were limited to choosing an expectant approach. Unfortunately, intrauterine death occurred only two weeks after diagnosis. Induction of labour indicated the presence of obvious umbilical vein varices, which were predominantly located near the placenta (Fig. 3a), with a large range of more than 20 cm in length and a widest diameter of approximately 6 cm (Fig. 3b, c). A histopathological examination revealed umbilical vein thrombosis (Fig. 3d).

## Discussion and conclusions

Here, we present a case of a foetus with the largest EAUVV ever observed. To date, no more than 20 cases of EAUVV have been reported. During pregnancy, EAUVVs can have a mean diameter of 3.4 cm (range: 1 to 5 cm) and most commonly occur on the foetal side of the umbilical vein; the median gestational age at delivery is 35 weeks (range: 32 to 41 weeks), and some foetuses experience intrauterine growth restriction, unstable foetal health, or death. To date, no chromosomal abnormalities have been associated with EAUVV [1].

Two-dimensional ultrasonography can be used to diagnose UVV prenatally, although three-dimensional ultrasonography can provide a more comprehensive picture [7]; MRI can also be used in conjunction with ultrasonography to assist in the diagnosis of UVV [8]. It should be noted, however, that small varices may escape prenatal detection. In many cases, the diagnosis is only confirmed postnatally or during an autopsy. This is because many smaller extra-abdominal UVVs look similar to umbilical cord cysts during ultrasonography, making it very possible to miss the diagnosis or make a mistake [9–11].

In this case, the extensive foetal EAUVV with blood stasis may have caused the thrombosis and the ensuing foetal death. In addition, thickened Wharton jelly and morphological abnormalities in the portion of the umbilical cord near the placenta were major risk factors for umbilical cord occlusion [5]. Umbilical vein thrombosis is uncommon, but it can pose a considerable health threat in some cases. According to the literature reviews, umbilical vein thrombosis is more commonly encountered than umbilical artery thrombosis (71–85% versus 11–15%). Thrombosis of the umbilical artery, however, is associated with poor foetal outcomes [12]. There are many possible causes of umbilical vein thrombosis, including mechanical, vascular, inflammatory, foetal, maternal, and iatrogenic causes [11]. Common complications of umbilical cord thrombosis include structural abnormalities of the umbilical cord, infection, preeclampsia, phlebitis, foetal anaemia, maternal diabetes, maternal coagulation disorders, and foeto-maternal haemorrhage [1, 5, 12]. The umbilical cord should be scanned carefully if the patient has any of the above complications. If the umbilical cord is too long, blood stasis is likely to occur due to cord compression (due to factors such as umbilical cord knots), resulting in thrombosis of the umbilical vessels; if the umbilical cord is too short, vessel stretching is more likely to occur during childbirth, which leads to vascular injury and thrombosis [13, 14].

Since UVVs (especially massive varices) increase the risk of thrombosis, foetuses should be closely monitored if abnormalities are discovered [11, 15, 16]. Follow up should be performed with increased frequency to diagnose the appearance of turbulent flow in the UVV, and exclude signs of cardiac failure, such as hydrops foetalis [17]. No consistent results regarding whether affected pregnancies should be delivered immediately are reported in the current literature. Some consider labour induction once foetal lung maturity is confirmed or with the appearance of signs of foetal distress (including the possible demonstration of thrombosis within the umbilical cord) [18]. The delivery of a full-term healthy foetus was reported in the literature; however, severe foetal bradycardia occurred during delivery. This suggests that umbilical vein thrombosis can substantially increase the foetal risk associated with natural childbirth [19]. Elective caesarean sections have been considered safer and more effective in late pregnancy [5]. UVV was diagnosed in this case at only 25 weeks and 3 days of gestation. The condition of the foetus was explained in detail to the patient. Because the foetus had obvious EAUVV and blood stasis, thrombosis could occur at any time, potentially resulting in foetal hypoxia or even death. According to the patient, after consulting a neonatologist, she considered the burden of hospitalization for an early delivery to be too great, and expressed concern about the possible complications associated with a preterm birth. The patient was unwilling to bear the possible risks associated with a premature birth and also refused to be hospitalized. As a result, we were left with expectant therapy as the only treatment option. The foetus, however, died two weeks after diagnosis because of the wide range and rapid aggravation of the varices. Other patients, however, may elect to deliver a living foetus to maximize survival and accept the risks related to prematurity. In such circumstances, it would be necessary to perform Doppler monitoring early to confirm the accuracy of the diagnosis. When making a clinical decision, it is important to comprehensively consider the extent and complications of UVV, gestational age, foetal haemodynamics, and other relevant conditions. We recommend close monitoring with hospital admission (to facilities capable of handling extreme preterm foetuses) after variability in delivery for worsening haemodynamic status.

## Supplementary Information

**Additionalfile 1. **The vertical section showedthat the broadened umbilical vein was approximately 20 cm long. Two-dimensionalultrasound suggested blood stasis within the varix.

## Data Availability

All data generated or analysed during this study are included in this published article [and its supplementary information files].

## Abbreviations

EAUVV: Extra-abdominal umbilical vein varix
MRI: Magnetic resonance imaging
UVV: Umbilical vein varix
PSV: Peak-systolic velocity
